# Direct-view endoscopic ultrasound-guided fibrotic hepaticojejunostomy stricture managed with a modified stent tube

**DOI:** 10.1055/a-2762-8142

**Published:** 2026-01-20

**Authors:** Koichi Soga, Mayumi Yamaguchi, Masaru Kuwada, Ryosaku Shirahashi, Ikuhiro Kobori, Masaya Tamano

**Affiliations:** 1 26263 Department of Gastroenterology, Dokkyo Medical University Saitama Medical Center, Koshigaya, Japan

Biliary anastomotic strictures in surgically altered anatomies (SAAs) are challenging late complications, especially when involving the posterior segmental bile duct (PSBD). While balloon-assisted enteroscopy–endoscopic retrograde cholangiopancreatography (BE-ERCP) is the standard treatment, its effectiveness can be hindered by long afferent limbs, sharp angulations, and dense fibrosis at hepaticojejunostomy sites. When BE-ERCP or percutaneous rendezvous fails, endoscopic ultrasound-guided biliary drainage (EUS-BD) represents an alternative 1 . A direct-view convex EUS-BD (DV-EUS-BD) is suitable for SAAs, as its stable forward view, achieved through precise real-time ultrasonography, enhances device manipulation and puncture accuracy. However, advancing stents across fibrotic anastomoses remains challenging. We successfully used a tapered-tip modified single pig-tail plastic stent (mSPPS) derived from an endoscopic nasobiliary drainage tube (ENBD) 2 3 , following DV-EUS-BD.

A 72-year-old female patient had undergone pancreaticoduodenectomy for a serous cystic neoplasm ~6 years before her referral. Three years before, she experienced recurrent fever and right upper quadrant pain. Imaging showed the localized dilatation of the PSBD, suggesting a post-surgical stricture. Several BE-ERCP attempts failed to achieve ductal access. Four months before referral, she developed fever, elevated inflammatory markers, and a liver abscess. The rendezvous approach failed during percutaneous transhepatic cholangial drainage as the guidewire deviated through a fibrotic anastomosis ( Fig. 1 , Fig. 2 ).

**Fig. 1:**
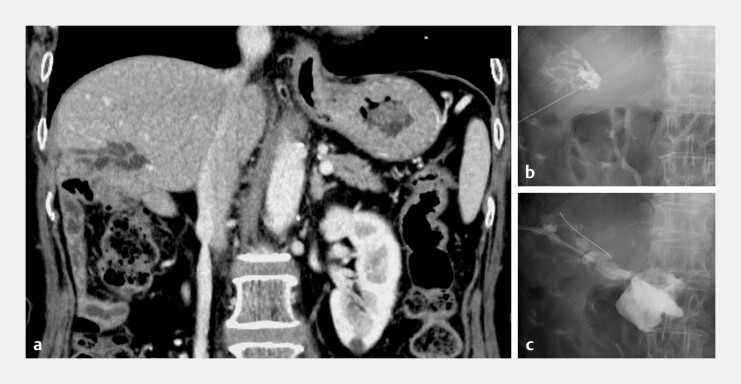
Imaging and interventional findings during attempted relief of hepaticojejunostomy strictures at the patient’s previous hospital. **a** Contrast-enhanced abdominal computed tomography (CT) demonstrating localized dilatation of the posterior segmental bile duct that was associated with repeated episodes of cholangitis. **b, c** Percutaneous transhepatic cholangial drainage (PTCD) performed at the previous hospital. Because of a severely fibrotic hepaticojejunostomy stricture, the guidewire and devices penetrated into the retroperitoneal cavity, resulting in the failure of PTCD.

**Fig. 2:**
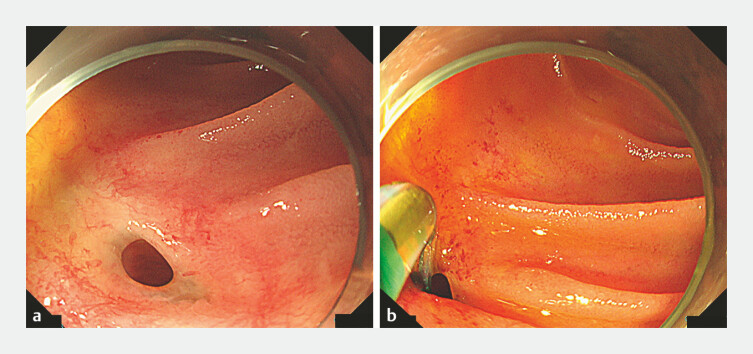
Attempted enteroscopic biliary drainage at our center following the patient’s referral. **a** Single balloon-assisted enteroscopy revealed a single orifice at the hepaticojejunostomy site. **b** Contrast injection from the anastomotic site failed to visualize the posterior segmental bile duct (PSBD). Selective access to the PSBD was attempted using an endoscopic sphincterotomy knife and guidewire manipulation along the presumed bile duct axis, but was unsuccessful.

At her referral, DV-EUS-BD facilitated the secure identification and puncture of the PSBD with a 22-G needle, confirmed via aspiration and cholangiography. Plastic stent placement was unsuccessful, owing to severe fibrosis. However, a tapered-tip 7.5-Fr mSPPS (Flexima, Boston Scientific Corporation, MA, USA) was advanced smoothly across the jejunal limb, hepatic parenchyma, and ductal wall ( Fig. 3 , Fig. 4 ). It was deployed without complications, produced adequate drainage ( Fig. 5 ), and was maintained until an elective replacement 3 months later ( Video 1 ).

**Fig. 3:**
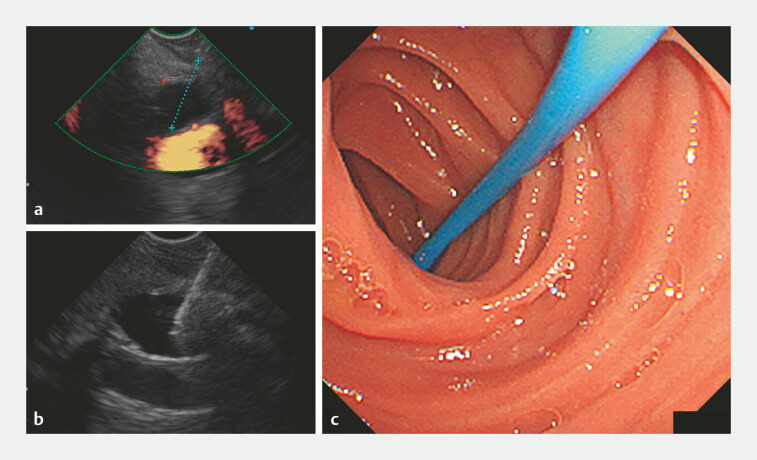
Endoscopic ultrasound-guided hepaticojejunostomy (EUS-HJS) using a direct-view convex EUS. **a** The posterior segmental bile duct (PSBD) visualized under EUS with doppler, confirming the presence of adjacent vasculature. **b** A 22-G fine needle was advanced into the PSBD under EUS guidance. **c** The PSBD wall was found to be markedly fibrotic because of repeated cholangitis, making standard plastic stent insertion unsuccessful. A tapered-tip endoscopic nasobiliary drainage tube (7.5-Fr) was therefore trimmed to a straight 20-cm segment and deployed into the PSBD, achieving successful drainage.

**Fig. 4:**
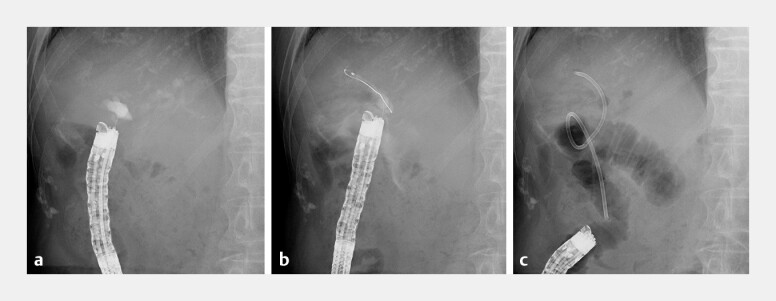
Fluoroscopic images of EUS-HJS using a direct-view convex EUS. **a** Visualization and puncture of the posterior segmental bile duct (PSBD) with a 22-G needle under EUS guidance, confirming ductal access. **b** Guidewire insertion through the puncture tract into the PSBD. **c** Plastic stent insertion was unsuccessful because of severe fibrosis from repeated cholangitis. A 7.5-Fr tapered-tip endoscopic nasobiliary drainage tube was therefore trimmed to a 20-cm straight segment, advanced along the guidewire, and successfully deployed into the PSBD, achieving effective drainage. EUS-HJS, endoscopic ultrasound-guided hepaticojejunostomy.

**Fig. 5:**
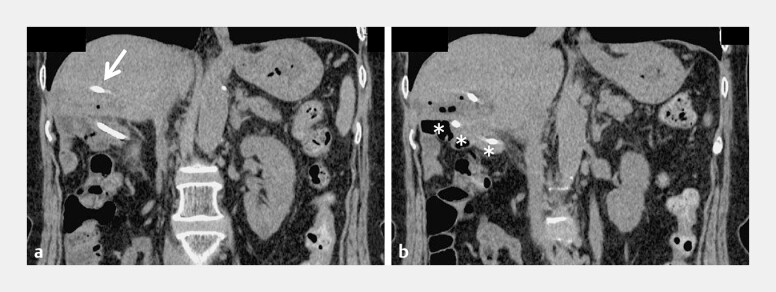
Follow-up abdominal CT performed 1 week after EUS-guided hepaticojejunostomy. **a** Abdominal CT demonstrating the correct placement of the modified endoscopic nasobiliary drainage tube, with its tip located in the posterior segmental bile duct (arrow). **b** The tube was observed to traverse the punctured tract from the bile duct to the jejunal limb (asterisk), confirming an appropriate drainage route and position. CT, computed tomography; EUS, endoscopic ultrasound.

Successful management of a fibrotic hepaticojejunostomy stricture in a surgically altered anatomy using direct-view endoscopic ultrasonography and a modified tapered single pig-tail plastic stent tube.Video 1

This case highlights the complementary values of DV-EUS-BD for accessing SAAs and mSPPS for overcoming rigid strictures, representing a safe and effective option for complex postoperative biliary obstruction.

Endoscopy_UCTN_Code_TTT_1AS_2AH

## References

[1] Siripun A Sripongpun P Ovartlarnporn B Endoscopic ultrasound-guided biliary intervention in patients with surgically altered anatomy World J Gastrointest Endosc 2015 7 283 289 10.4253/wjge.v7.i3.283 25789101 PMC4360449

[2] Soga K Single-pigtail plastic stent made from endoscopic nasobiliary drainage tubes in endoscopic ultrasound-guided gallbladder drainage: a retrospective case series Clin Endosc 2024 57 263 267 10.5946/ce.2022.213 37011902 PMC10984743

[3] Soga K Mukai H Kitae H Management of afferent loop obstruction using multiple single-pigtail plastic stents in a patient with recurrent metastatic pancreatic cancer Endoscopy 2022 54 E1041 E1042 36007911 10.1055/a-1907-4589 PMC9737439

